# Datura Stramonium an Emmenagogue

**Published:** 1848-04

**Authors:** B. F. Jones

**Affiliations:** St. Louis county, Missouri


Art. II.—*Datura Stramonium an Emmenagogue.* By B. F. Jones, M.D., of St. Louis county, Missouri.

Distinguished practitioners of medicine doubt whether there be any medicine known, capable of so influencing the uterus, as to compel it, even when healthy in other respects to secrete the catamenia. Hippocrates made no pretensions to a knowledge of any such medicine, nor did Sydenham; and Boerhaave's catalogue of emmenagogues comprehends only such medicines as are conspicuous for determining to the pelvic viscera—stating that "we have no uterine remedies but sudorific determined to the uterus." Dr. Eberle tells us, "It is very doubtful whether any of the articles which have hitherto been employed for this purpose possess any direct influence over the uterine secretions;" and such, I presume, is the experience of physicians generally.

In reasoning upon this subject, it occurred to me, that inasmuch as there was a medicine (*the secale cornutum*) capable of exerting a specific impression upon the uterus, and forcing it to the energetic performance of *one* function—the expulsion of the ovum—there was nothing unphilosophical in the hope that a remedy might exist capable of urging upon it the elaboration of *another*—the secretion of the catamenia.

If nature, in the exuberance of her munificence, had vouchsafed to us a medicine endowed with capability to abridge the parturient effort, it was scarcely expecting too much from her beneficence to hope that among her rich *arcana* was another capacitated to place the uterus in a condition for the exercise of its function of reproduction. In the absence of one, the other would not unfrequently be useless—and to that extent mar that beautiful harmony, and adaptation of means to ends, of which nature is so observant in all her operations.

Persuaded of the philosophy of this view, as far back as 1839, when I practiced my profession in the city of Chihuahua, in Mexico, I was led to experiment with a great number of powerful plants, of recognized capability strongly to impress the organism, with the hope of discovering among them a special relevancy of action for the uterus, during the suspension of its periodical function. The datura stramonium, from the energy it displays in affecting the nervous system, without exciting the circulation, together with its simultaneous relaxing effect upon the bowels, with the increase of urinary and dermoid secretion, presented strong claims to my attention in this experimental inquiry.

The result of my investigation is the belief that stramonium does possess the power of imposing upon the uterus a renovated secretory effort, to the same extent that mercury does upon the liver, or the salivary glands, *ceteris paribus*, and is therefore eminently entitled to the cognomen of an emmenagogue; a distinction that cannot of right be conferred upon any other known article in the materia medica. So that properly there belongs to each of the important classes of abortives and emmenagogues, but one article—secale cornutum, and datura stramonium.

I will endeavor to illustrate the above views by the notes of a single case, which, though the most prominent, is by no means isolated upon my case-book.

Mrs. K., æt. 31, in the spring of 1837, had a violent attack of congestive fever, for which she was salivated. I was called to see her October 1st, 1841, four years after the attack of fever, for the first time, to prescribe for a suppressed menstruation, which she informed me, had existed for four years. She had been in the hands of numerous physicians, who had rung the changes on all the emmenagogues, remedies local and constitutional, known to the books, or the tradition of the "mothers." Her general health was miserable, and a thousand and one nervous phenomena, inseparably associated with a protracted suspension of uterine secretion, made existence a burden. I gave her four of the following pills:

℞. Prot. chlo. hydg.
P. rhei opt. ää. gr. xxiv.
Gm. gamboge, gr. viij.
Acacia mucilaginis, q. s. ut fiant pil. ponder. gr. v.

After the operation of the pills, I put her on the tinct. semen. stramonii, prepared by the following recipe:

℞. Semen stramonii uncias iv.
Alcoholis diluti octantem unum.
Degere per dies decem, et per chartam cola.

I directed her to take twenty drops, three times a day, for the first day, adding a drop to the dose each day, and to continue it, either until it produces dizziness, vertigo, *or the catamenia.* In ten days after I first saw her, I was summoned to her again, and learned that the day previous she had commenced evacuating blood from the bowels, in small quantities and at irregular intervals. I gave it as my opinion that this was a vicarious evacuation, and the harbinger of a regular catamenia *per vias naturales;* and directed her to suspend the medicine for two weeks, and then resume it again in the same dose last taken, (xxx. qtt., *ter die,*) to be increased as before directed, and to report to me in two weeks thereafter. At the expiration of the two weeks from the resumption of the medicine, she reported a regular and plentiful catamenia. From this time she was perfectly regular, until she became pregnant, and in due course of time was delivered of a healthy child. She entirely regained her health, and has subsequently borne two other children.

St. Louis County, Mo., Dec. 12, 1847.

